# Clinical application of “Double R” anastomosis technique in laparoscopic pancreaticoduodenectomy procedure

**DOI:** 10.1097/MD.0000000000026204

**Published:** 2021-05-28

**Authors:** Wei Tang, Jian-Guo Qiu, Gui-Zhong Li, Yu-Fei Zhao, Cheng-You Du

**Affiliations:** a Department of Hepatobiliary Surgery, The First Affiliated Hospital of Chongqing Medical University; b Department of General Surgery, The Affiliated Chongqing Beibei Traditional Chinese Medical Hospital of Guangzhou University of Chinese Medicine, Chongqing, China.

**Keywords:** “Double R” technique, laparoscopic pancreaticoduodenectomy, novel anastomosis, pancreatic fistula, pancreaticojejunostomy, Whipple procedure

## Abstract

Laparoscopic pancreaticoduodenectomy (LPD) is widely used as a treatment for periampullary tumors and pancreatic head tumors. However, postoperative pancreatic fistula (POPF), which significantly affects mortality and length of hospital stay of patients, remains one of the most common and serious complications following LPD. Though numerous technical modifications for pancreaticojejunostomy (PJ) have been proposed, POPF is still the “Achilles heel” of LPD.

To reduce POPF rate and other postoperative complications following LPD by exploring the best approach to manage with the pancreatic remnant, a novel duct-to-mucosa anastomosis technique named Double Layer Running Suture (Double R) for the PJ was established. During 2018 and 2020, a totally 35 patients who underwent LPD with Double R were included, data on the total operative time, PJ duration, estimated blood loss, recovery of bowel function, postoperative complications, and length of hospital stay were collected and analyzed.

The average duration of surgery was (380 ± 69) minutes. The mean time for performing PJ was (34 ± 5) minutes. The average estimated blood loss was (180 ± 155) mL. The overall POPF rate was 8.6% (3/35), including 8.6% (3/35) for the biochemical leak, 0% (0/35) for Grade B, and 0% (0/35) for Grade C. No patient suffered from biliary fistula, post-pancreatectomy hemorrhage, and intra-abdominal infection, the 30-day mortality was 0%.

Double R anastomosis is potentially a safe, reliable, and rapid anastomosis with a low rate of POPF and post-pancreatectomy hemorrhage. It provides surgeons more options when performing LPD. However, its safety and effectiveness should be verified further by a larger prospective multicenter study.

## 1 Introduction

Pancreaticoduodenectomy (PD) remains the standard surgical procedure for periampullary tumors and pancreatic head tumors and is viewed as one of the most challenging procedures in abdominal surgeries. As the development of the concept of minimally invasive surgery, in 1994, laparoscopic PD (LPD) was first aroused.^[1]^ Compared with open PD, LPD was thought to be associated with a shorter length of hospital stay, lower estimated blood loss, and comparable oncologic outcomes and long-term overall survival.^[2–4]^ The postoperative complications morbidity associated with LPD ranged from 30.0% to 50.0%.^[5–6]^ Among all of the complications following LPD, postoperative pancreatic fistula (POPF) is considered the most common and serious one, which significantly affects mortality and length of hospital stay of patients for lethal abdominal bleeding and infection caused by it.^[7–8]^ The occurrence of POPF was related to 3 important risk factors, which included patient factors (age, sex, level of jaundice, and others), operation factors (operation time, blood loss, type of anastomosis, stenting of anastomosis, and drainage management), and pancreas factors (pancreatic texture, fatty pancreas, pancreatic duct size, blood supply of the cut end, original pathology, and others).^[9–11]^ As reported by previous studies,^[12–14]^ POPF occurred in 2% to 28.8% of the cases receiving LPD and the mean mortality of Grade C POPF was up to 25.7%. Thus, POPF has commonly been viewed as the “Achilles heel” of LPD. Currently, Blumgart anastomosis is considered the mainstay of pancreatic anastomosis and is thought to be simple, safe, and reliable, whereas its effectiveness in patients with small pancreatic duct caliber remains the weakness.^[15–16]^ Therefore, improvement and innovation of the way of pancreatic anastomosis are still the tasks for pancreatic surgeons. As a result, numerous surgical methods, including end-to-end invaginated anastomosis, end-to-side invaginated anastomosis, duct-to-mucosa anastomosis, and their modifications have been proposed and applied for treatment options.^[17]^ Nevertheless, no consensus was reached on the best approach to anastomose pancreas following LPD. Thus, with the aim of simplifying the procedure of reconstruction, shortening the time of operation, improving the quality of the pancreaticojejunostomy (PJ), and providing more options for pancreatic surgeons, we developed a new simple and safe duct-to-mucosa anastomosis technique named Double Layer Running Suture (Double R) anastomosis technique for the PJ procedure during our clinical practice.

## 2 Methods

### 2.1 Demographic data of patients and preoperative evaluation

Our first case of LPD was performed in 2015 at the Department of Hepatobiliary Surgery of the First Affiliated Hospital of Chongqing Medical University. To date, more than 400 cases of LPD, most of which used duct-to-mucosa anastomosis, have been performed at our center. From November 2018, the LPD procedure with Double R anastomosis technique began to be performed. Till January 2020, a total of 35 patients who underwent LPD with Double R anastomosis were included in this single-center retrospective study and all cases were consecutive without any selection criterion. As shown in Table 1, among these patients, 16 were male and 19 were female. The included patients’ ages ranged from 36 to 72 years, mean age was (59.0 ± 11.0) years. Body mass index ranged from 18 to 28 kg/m^2^ and mean body mass index was (23.2 ± 4.4) kg/m^2^. According to the Eastern Cooperative Oncology Group performance status score, 29 of the 35 patients got 0 to 1 scores and 6 got 2 scores. Of the 35 patients, 25 were classified as American Society of Anesthesiology level I, 7 were level II, and 3 were level III. All patients underwent contrast-enhanced computed tomography or magnetic resonance imaging, and a CA19-9 cancer antigen assay preoperatively to confirm the clinical diagnosis, evaluate the extent of the disease, and confirm the resectability of the tumor. Other routine examinations included blood test, liver and renal function test, coagulation function test, electrocardiogram, chest X-ray, and so on. Pathological examination of the specimen was also performed after surgery. This study was approved by the Ethics Committee of the First Affiliated Hospital of Chongqing Medical University and the written informed consent was obtained from the patients. This study was also registered with the Open Science Framework platform and was available at osf.io/2erwq.

**Table 1:** Demographic data of patients.

Variables	
Age	59.0 ± 11.0 years
Sex (male/female)	16/19
Body mass index	23.2 ± 4.4 kg/m^2^
Eastern Cooperative Oncology Group (n, %)	
0∼1	29 (82.9%)
2	6 (17.1%)
American Society of Anesthesiology (n, %)	
I	25 (71.4%)
II	7 (20.0%)
III	3 (8.6%)
Pathological diagnosis (n, %)	
Pancreatic ductal adenocarcinoma	6 (17.1%)
Distal cholangiocarcinoma	10 (28.6%)
Adenocarcinoma of the duodenum	12 (34.3%)
Metastatic clear cell renal cell carcinoma of pancreatic head	2 (5.7%)
Chronic pancreatitis	2 (5.7%)
Serous cystadenoma	3 (8.6%)

### 2.2 Surgical technique

The patients were placed in the supine position with legs apart and an anti-Trendelenburg position (15°–30°) was used. After general anesthesia, a pneumoperitoneum with a pressure of 12 to 14 mmHg CO_2_ was established. One 10 mm trocar for the laparoscopy port was inserted below the umbilicus. Separately, one 12 mm trocar and one 10 mm trocar was inserted at the right and left midclavicular line, 3 to 4 cm above the umbilicus. Two 5 mm trocars were inserted at the right and left anterior axillary line under the costal margin.

All operations were performed by a fixed team and no pylorus-preserving procedure was performed in any of the included patients. After the resectability was ascertained, resection of the pancreatic head with the adjacent duodenum was performed in a standard fashion.^[18]^ In this case, the malignant tumor was inseparable from the vein, wedge, or segmental resection of the vein was performed. Lymph nodes around the pancreatic head, the hepatoduodenal ligament, the common hepatic artery and the celiac trunk, and the superior mesenteric artery were all dissected. Then, the free end of the jejunum was brought up through the root of the transverse mesocolon to the supramesocolic compartment. After the pathological specimen was removed, Double R duct-to-mucosa anastomosis was performed.

**Figure 1:**
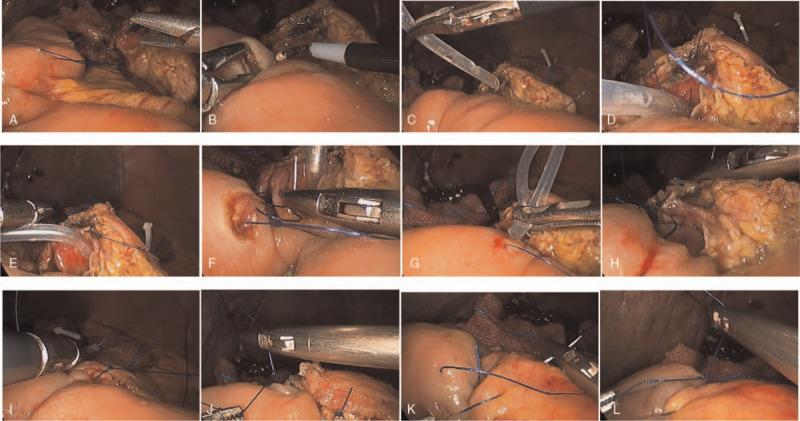
Color pictures of Double Layer Running Suture anastomosis procedure (A) The seromuscular layer of the dorsal jejunum and the dorsal 0.5–1.0 cm pancreatic tissue from the resection margin were sutured from head to foot by a horizontal mattress running suture (suture A). (B) A hole corresponding to the pancreatic duct was created in the jejunum. (C) to (E) A plastic catheter was inserted as an internal stent into the main pancreatic duct and was secured with a 4–0 or 5–0 absorbable suture which went through the catheter and the posterior wall of the main pancreatic duct. (F) The anastomosis of the posterior wall of the main pancreatic duct and the jejunal mucosa was performed from head to foot by a running suture with a 4–0 or 5–0 Prolene suture (suture B). (G) Another end of the catheter was inserted into the hole in the jejunum. (H) The anastomosis of the anterior wall of the main pancreatic duct and the jejunal mucosa was performed from head to foot with a 4–0 or 5–0 Prolene running suture (suture C). (I) and (J) The knots of the head side and foot side of suture B and C were tied separately. (K) The seromuscular layer of ventral jejunum and the ventral 0.5–1.0 cm pancreatic tissue from the resection margin were sutured from foot to head by a horizontal mattress running suture with suture A. (L) The knot of suture A was tied.

**Figure 2:**
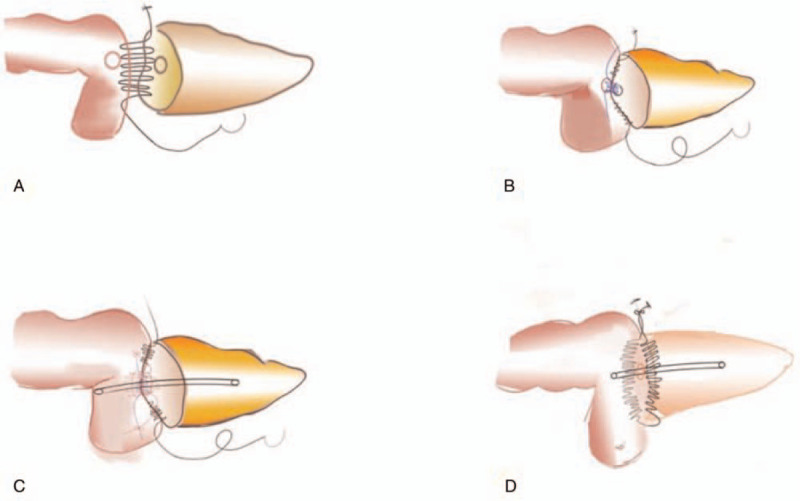
Mode chart of Double Layer Running Suture anastomosis procedure. (A) The seromuscular layer of the dorsal jejunum and the dorsal 0.5–1.0 cm pancreatic tissue from the resection margin were sutured from head to foot by a horizontal mattress running suture (suture A). (B) The anastomosis of posterior wall of the main pancreatic duct and the jejunal mucosa was performed from head to foot by a running suture with a 4–0 or 5–0 Prolene suture (suture B). (C) The anastomosis of the anterior wall of the main pancreatic duct and the jejunal mucosa was performed from head to foot with a 4–0 or 5–0 Prolene running suture (suture C). (D) The seromuscular layer of ventral jejunum and the ventral 0.5–1.0 cm pancreatic tissue from the resection margin were sutured from foot to head by a horizontal mattress running suture with suture A.

Double R procedure was an anastomosis technique of running sutures of both ventral and dorsal part of pancreatic parenchyma and a seromuscular layer of the jejunum, and anterior and posterior wall of the main pancreatic duct and jejunal mucosa. After the pancreatic stump remnant was dissected to approximately 1.0 to 2.0 cm using an ultrasonic scalpel, the suture of the dorsal part of the pancreatic parenchyma and seromuscular layer of the jejunum was firstly performed. With a 3–0 or 4–0 Prolene suture (suture A), the seromuscular layer of dorsal jejunum and the dorsal 0.5 to 1.0 cm pancreatic tissue from the resection margin were sutured from head to foot by a horizontal mattress running suture (Figs. 1Aand Fig. 2A). Without tightening up the suture and tying the knot for the first and last stitches, the tails of the suture were clipped with titanium clips to prevent its slippage. A hole corresponding to the pancreatic duct was created in the jejunum using an electronic coagulator or an ultrasonic scalpel (Fig. 1B) and a plastic catheter of which the diameter was matched with the caliber of pancreatic duct was prepared with 3to 5 side holes in its one end. Then, the end with side holes was inserted as an internal stent into the main pancreatic duct (Fig. 1C). After the catheter was secured with a 4–0 or 5–0 absorbable suture which went through the catheter and the anterior wall of the main pancreatic duct (Fig. 1D and 1E), the anastomosis of the posterior wall of the main pancreatic duct and the jejunal mucosa was performed from head to foot by a running suture with a 4–0 or 5–0 Prolene suture (suture B) and without tightening up the suture and tying the knot (Figs. 1F and Fig. 2B). Then another end of the catheter was inserted into the hole in the jejunum (Fig. 1G). Subsequently, the anastomosis of the anterior wall of the main pancreatic duct and the jejunal mucosa was performed from head to foot with a 4–0 or 5–0 Prolene running suture (suture C) (Figs. 1H and 2C). After suture A, B, and C were tightened up and the distance between the pancreatic stump and the jejunum was shortened, the knots of the head side and foot side of suture B and C were tied separately (Fig. 1I and 1J). Then, with suture A, the seromuscular layer of ventral jejunum and the ventral 0.5 to 1.0 cm pancreatic tissue from the resection margin were sutured from foot to head by a horizontal mattress running suture (Fig. 1K). Lastly, after tightening up suture A again, the knot was tied and Double R anastomosis was finished (Figs. 1L and 2D).

Further reconstruction of gastrointestinal continuity included end-to-side hepaticojejunostomy and antecolic gastrojejunostomy. Hepaticojejunostomy was performed 10.0 to 15.0 cm from the PJ and antecolic gastrojejunostomy was performed approximately 50.0 cm from the PJ. A nasogastric tube was placed in all patients. Operative drains were placed in the dorsal of the hepaticojejunostomy and superior and inferior of the PJ.

### 2.3 Postoperative treatment and surveillance

Generally, the nasogastric tube was left for 2 to 3 days. Parenteral nutrition was used for 3 to 4 days and then oral feeding was used. Other postoperative routine treatments included antibiotic, somatostatin, proton pump inhibitor, hemostatic drug, and so on. Characteristics and volume of the drains were recorded daily, and drainage samples were sent for amylase and lipase levels tests on day 1, 3, 5, and 7 after surgery. The removal of drains depended on the levels of amylase and lipase and the volume of drainage. A routine computed tomography scan was performed 7 days after surgery to evaluate intra-abdominal conditions. Other routine examinations included blood test, liver and renal function test, coagulation function test, and so on.

### 2.4 Outcome of interest and definition

Totally 11 outcomes were retrospectively collected and analyzed, including total operation time, PJ duration, estimated blood loss, recovery of bowel function, postoperative complications (POPF, biliary fistula, post-pancreatectomy hemorrhage, intra-abdominal infection, delayed gastric emptying, and mortality), and length of hospital stay.

Postoperative severe complications were defined according to the Clavien–Dindo classification of surgical complications.^[19]^ The definition of POPF was a drainage of fluid on or after postoperative day 3 with an amylase level greater than 3 times the serum amylase level and POPF was classified into 3 grades (biochemical leak, grade B, and grade C) according to the international grading system.^[7]^ Biliary fistula was defined as bile contents (more than 10 mL/day, last for at least 5 days) in the abdominal drains or leakage found at relaparotomy. Post-pancreatectomy hemorrhage was defined and classified by 3 parameters: time of onset, location, and severity of hemorrhage according to the International Study Group of Pancreatic Surgery.^[20]^ Intra-abdominal infection was defined as clinical signs (body temperature higher than 38.5°C combined with leukocyte count higher than 12 × 10^9^/L) and the presence of an intra-abdominal abscess. Delayed gastric emptying was defined as gastric stasis requiring nasogastric intubation for at least 7 days or the reinsertion of a nasogastric tube after the failure of postoperative oral feeding.^[21]^ Postoperative death was defined as death that was associated with the operation and occurred within 30 days postoperatively.

## 3 Results

As shown in Table 2, the average duration of surgery was (380 ± 69) minutes and the mean time for performing PJ was (34 ± 5) minutes. The average estimated blood loss was (180 ± 155) mL. The mean caliber of the pancreatic duct was (3.1 ± 1.3) mm. Of the 35 patients, 16 were with soft pancreas, 10 were with hard pancreas, and 9 were with moderate pancreas. The average time to the first passage of flatus postoperatively was (2.2 ± 0.8) days. The mean time to start the liquid diet and semi-liquid diet was (3.5 ± 1.1) days and (5.5 ± 0.7) days, respectively. The average hospital stay of the 11 patients was (14 ± 10) days. According to the postoperative pathological diagnoses (shown in Table 1), 30 patients were diagnosed with malignant diseases, including pancreatic ductal adenocarcinoma in 6 patients, distal cholangiocarcinoma in 10 patients, adenocarcinoma of the duodenum in 12 patients, and metastatic clear cell renal cell carcinoma of pancreatic head in 2 patients. Five patients were diagnosed with benign diseases, including chronic pancreatitis in 2 patients and serous cystadenoma in 3 patients. Of the 35 patients, 2 were diagnosed abdominal lymph nodes metastasis. Totally 3 times surgical complications occurred in 2 patients postoperatively, including delayed gastric emptying and gastrojejunostomy anastomosis bleeding in 1 patient (Clavien I), and respiratory tract infection in another patient (Clavien II). All of these patients were managed with non-operative treatments and went to full recovery. According to the international grading system,^[7]^ POPF occurred in 3 patients (8.6%) (all were diagnosed with adenocarcinoma of the duodenum) and all of them were biochemical leak (8.6%), no patient suffered from grade B (0%) or grade C (0%) POPF. Except for the high amylase level in the drainage, both patients had no abdominal symptoms and signs and went to full recovery after adequate drainage. No patients suffered from biliary fistula, post-pancreatectomy hemorrhage, and intra-abdominal infection. The 30-day mortality of our case series was 0%.

**Table 2:** The postoperative details and surgical outcomes.

Variables	
Total operative time	380 ± 69 min
Pancreaticojejunostomy duration	34 ± 5 min
Estimated blood loss	180 ± 155 mL
Time to first passage of flatus	2.2 ± 0.8 days
Postoperative hospital stay	14 ± 10 days
Complications (n, %)	
Pancreatic fistula	3 (8.6%)
Biochemical leak	3 (8.6%)
Grade B	0 (0%)
Grade C	0 (0%)
Bile leakage	0 (0%)
Delayed gastric emptying	0 (0%)
Post-pancreatectomy hemorrhage	0 (0%)
Intra-abdominal infection	0 (0%)
Death within 30 days postoperatively	0 (0%)

## 4 Discussion

LPD has been viewed as a safe and feasible procedure as the improvement of surgical experience. Nowadays, the mortality after an LPD was a low of about 5.0% in experienced pancreatic centers. However, the overall postoperative complication morbidity remains at a high level of 30.0% to 50.0%.^[5–6]^ Among all complications, POPF is considered the most common and serious one after LPD, which might lead to lethal abdominal bleeding and infection. And it is also considered the leading cause of death postoperatively.^[22]^ Related studies indicated the main risk factors of POPF included pancreatic texture, pancreatic duct size, and type of anastomosis.^[8,23]^ In our study, all POPF patients were diagnosed with adenocarcinoma of the duodenum, the pancreatic texture was soft and the pancreatic duct was small in them. These might be the key factors leading to POPF in our case series. To reduce the POPF rate, approximately 50 PJ techniques have been proposed over the past decades in the management of the pancreatic remnant. However, none of these techniques could avoid POPF completely. Previous studies showed the POPF rates of end-to-end invaginated anastomosis, end-to-side invaginated anastomosis, and duct-to-mucosa anastomosis were 11.7%, 16.5%, and 11.5%, respectively.^[24]^ Although the duct-to-mucosa technique is considerably more difficult to perform than invaginated anastomosis and the POPF rate of duct-to-mucosa technique is not superior to that of invaginated anastomosis,^[25]^ the duct-to-mucosa technique is still widely applied for that it is beneficial for the healing of PJ anastomosis and the coverage of the seromuscular layer of the jejunum in the resection margin of the pancreas may prevent bleeding from the pancreatic stump remnant. Furthermore, with the help of the magnification effect of laparoscopy when performing LPD, the application of the duct-to-mucosa technique becomes more feasible even in patients with small pancreatic ducts.

The mechanisms of POPF ascribe to the following aspects: (1) simple pancreatic leakage from the pinholes produced when stitches go through the capillary duct of pancreas and (2) digestive juice fistula from the weak spots of the anastomosis.^[26]^ Based on the mechanisms of POPF, an efficient PJ is considered to have the following advantages: (1) simple and safe; (2) abundant blood supply in the anastomosis; (3) effective hemostasis; (4) well tissue viability; (5) proper tension in sutures; and (6) accurate stitching.^[27]^ Generally, the way which conformed to the biological structure of the digestive tract the most was considered the best method of reconstruction of gastrointestine. Double R anastomosis is a type of duct-to-mucosa running suture PJ. Compared with interrupted sutures, running sutures could dramatically decrease the knotting times (totally 3 times during PJ procedure) and shorten the operation time. The separate running sutures of the anterior and posterior wall of the main pancreatic duct and jejunal mucosa could prevent the immoderate level of tension in the anastomosis especially when the pancreatic duct is small, thus avoid anomalous anastomotic blood circulation, and keep the diameter of the anastomosis at its largest level. Running suturing could also avoid the retention of pancreatic juice by the uniform tightening of the space between the jejunal wall and pancreatic stump remnant without leaving any dead space. Contrarily, the interrupted suture is time-consuming and easier to damage the pancreatic parenchyma. Moreover, untied interrupted sutures are more likely to be intertwined during operation. However, if every stitch is knotted, a previously tied stitch might shorten the distance between the jejunum and pancreatic stump remnant and thus hinder the next stitch. Furthermore, additional advantages of Double R anastomosis included the following: (1) it is consistent with the basic concept of duct-to-mucosa anastomosis; (2) the plastic catheter in the pancreatic duct could work as an internal stent as well as a drainage tube, and thus decreased the POPF rate; and (3) it is suitable for patients with different pancreatic duct sizes and pancreatic texture for its universal applicability. In our study, the incidence of POPF was low of about 8.6%, only 3 patients developed biochemical leak, no grade B or grade C POPF occurred. Therefore, fistula-related morbidity and mortality was largely avoided and were quite low in our case series using this new surgical technique.

However, we have to acknowledge several limitations in Double R anastomosis. First, the potential space between pancreatic remnant and jejunum, which is produced by the running sutures of the ventral and dorsal part of pancreatic parenchyma and jejunum, might be the breakthrough of POPF. Second, the requirement of surgical technique is high, especially in patients with small pancreatic ducts, and it could result in a higher learning curve for a surgeon. Third, this study was not a prospective, randomized study and all operations were performed by a single team, thus it might bring about some bias.

In conclusion, Double R is a potentially safe, reliable, and rapid PJ anastomosis technique associated with a low risk of POPF and postoperative hemorrhage. It provides surgeons more options when performing LPD and its clinical application is of value. However, the safety and effectiveness of this anastomosis should be verified further by a larger prospective multicenter study.

## Author contributions

Wei Tang and Jian-Guo Qiu performed the study and wrote the paper. Gui-Zhong Li and Yu-Fei Zhao collected and analyzed the data and made the tables and figures. Cheng-You Du designed the study, edited the manuscript, and offered suggestions for this study. Wei Tang and Jian-Guo Qiu contributed equally to this work.

**Conceptualization:** Wei Tang, Jian-Guo Qiu.

**Data curation:** Gui-Zhong Li, Yu-Fei Zhao.

**Formal analysis:** Wei Tang.

**Funding acquisition:** Cheng-You Du.

**Methodology:** Wei Tang, Jian-Guo Qiu.

**Project administration:** Cheng-You Du.

**Resources:** Cheng-You Du.

**Software:** Gui-Zhong Li, Yu-Fei Zhao.

**Supervision:** Cheng-You Du.

**Validation:** Cheng-You Du.

**Writing – original draft:** Wei Tang, Jian-Guo Qiu.

**Writing – review & editing:** Wei Tang, Jian-Guo Qiu, Cheng-You Du.
